# Removal efficiency of central vacuum system and protective masks to suspended particles from dental treatment

**DOI:** 10.1371/journal.pone.0225644

**Published:** 2019-11-26

**Authors:** Ming-Hui Liu, Chi-Tsung Chen, Li-Chuan Chuang, Wen-Ming Lin, Gwo-Hwa Wan

**Affiliations:** 1 Department of Pediatric Dentistry, Taoyuan Chang Gung Memorial Hospital, Taoyuan, Taiwan; 2 Graduate Institute of Clinical Medical Sciences, College of Medicine, Chang Gung University, Taoyuan, Taiwan; 3 Department of Pediatric Dentistry, Linkou Chang Gung Memorial Hospital, Taoyuan, Taiwan; 4 Department of General Practice Dentistry, Taoyuan Chang Gung Memorial Hospital, Taoyuan, Taiwan; 5 Department of Respiratory Therapy, College of Medicine, Chang Gung University, Taoyuan, Taiwan; 6 Department of Respiratory Care, Chang Gung University of Science and Technology, Chiayi, Taiwan; 7 Department of Obstetrics and Gynaecology, Taipei Chang Gung Memorial Hospital, Taipei, Taiwan; VIT University, INDIA

## Abstract

**Background:**

High levels of suspended particulate matters (PMs) and bioaerosols are created by dental procedures. The present study aimed to evaluate the size and concentration of PMs produced by drilling and grinding teeth, and to assess the efficiency of central vacuum system and protective masks for the removal of PMs.

**Methods:**

A total of 20 extracted permanent teeth were collected. A novel experimental system and particle counter were used to evaluate the PMs produced by dental procedures and the PM removal efficiency of a central vacuum system and surgical/N95 masks.

**Results:**

The number concentration of total PMs produced by drilling and grinding teeth was significantly higher than the indoor background concentration. The average aerodynamic diameter of particle was generally less than 1 μm. The average number concentration of ultrafine particles was 2.1x10^11^ particles/m^3^ during tooth drilling and grinding. The efficiency of the central vacuum system was 35.74% for PM_≥0.5_ and 35.41% for PM_10_. For PM_≥0.5_, the ratios of inside and outside masks were 0.8–1.34 without vacuum and 1.18–1.36 with vacuum. No difference was found with the use of surgical/N95 masks during dental therapy, with or without vacuum use.

**Conclusions:**

High levels of PMs were found during tooth drilling and grinding procedures, especially among PM_1_. The PM removal efficiency of a central vacuum system and surgical/N95 masks were limited.

## Introduction

Dental caries and periodontal disease are the most common dental diseases [1]. Common dental therapies for these diseases include tooth restoration or extraction procedures, dental scaling, and endodontic therapy. The main source of aerosols during dental therapy is the patient’s mouth, which can cause spread of microorganisms and affect the quality of dentistry environment [2]. Most dental procedures produce aerosols due to high-speed dental equipment usage or tooth scaling [2,3]. These aerosols can include bacteria such as *Streptococcus spp*., which has been detected in the air of the dental treatment room during dental therapy [4]. Even shortly following dental treatments, the dental treatment area retains a high concentration of airborne bacteria, which may spread to inactive dental treatment areas [5]. Infection control is critical to achieve healthy and effective dental treatments.

In addition to the routine use of standard barriers such as protective masks and gloves in the clinical setting, the universal use of pre-procedural rinses and high-volume evacuations is recommended to reduce infection risk [2,3]. During dental treatment procedures, dentists and all dental staff may expose to bioaerosols [4,5] and PMs [6–8]. Previous studies indicated that aerosols exposure was related to the risks of respiratory, liver, renal, and nervous systems dysfunction [9–11]. However, no study was evaluated the relationship between health effects of dental healthcare workers and aerosols exposure during dental treatment.

Few studies have assessed the size and concentration of particles produced by dentists during dental procedures such as tooth drilling. One study in the United States reported that dental procedures produced considerably more PM_0.3–0.5_ (particulate matter 0.3–0.5 μm in size) (2.32×10^8^–3.74×10^8^ particles/m^3^) than those that were present in the background air (2.22×10^8^ particles/m^3^) [6]. Others have reported higher PM concentrations during dental therapy procedures than during non-working periods (i.e., indoor background) [6,7,11–13].

Several steps can be taken to reduce aerosol contamination in the dental environment. First, the water line to dental chair equipment should be disinfected. Second, all dental treatment machines and instruments should be sterilized. Third, a valve and strainer should be installed in the treatment chair to prevent sucking reintegration infected liquid, aerosols, and PM. Personal protective equipment includes clothing, gloves, masks, and goggles, all of which are useful for infection control, may also be used [14]. The protective masks include surgical masks, N95 respirators, and surgical N95 respirators classified by the National Institute of Occupational Safety and Health (NIOSH) [15]. The surgical masks provide barrier protection against droplets, but do not prevent leakage around the edge of masks and then do not filter small airborne particles effectively [16]. The N95 respirators filter at least 95% of airborne particles with an aerodynamic diameter of 0.3-μm or above but are not resistant to oil particles.

Furthermore, patients can use mouthwash to disinfect the oral cavity prior to dental treatment, which can effectively reduce the spread and distribution of oral bacteria [3,14]. Previous studies have indicated that the use of rubber dams and high-power vacuum systems during treatment procedures may effectively reduce the concentration of aerosols [17,18]. A bench study also reported that using a conventional dental vacuum system (vacuum flow: 66 liters per minute, LPM) or high-volume evacuation system (vacuum flow: 360 LPM) during dental treatments can reduce fine and ultrafine particle concentrations [12].

Until recently, few studies have focused on the size and concentration of PMs produced by dental treatments. Thus, this study aimed to evaluate the size and concentration of suspended particles produced during tooth drilling and grinding, as well as assess the efficiency of a central vacuum system and protective masks for limiting PM production.

## Material and methods

### Tooth sample collection

The Human Trial Ethics Committee of Chang Gung Memorial Hospital approved the present study (IRB No: 201700408B0). This study recruited participants from Linkuo and Taoyuan Chang Gung Memorial Hospitals in Taiwan and all study subjects provided written informed consent. Study inclusion criteria consisted of a poor prognosis and extracted permanent teeth. We collected 20 tooth samples from recruited patients. Samples were teeth extracted by a professional dentist due to severe periodontal disease or deep caries. The occlusal surface of all samples was complete or only partially filled. All samples were placed in sterile distilled water following extraction rapidly.

### Experimental model for particle exposure assessment

**Fig 1:**
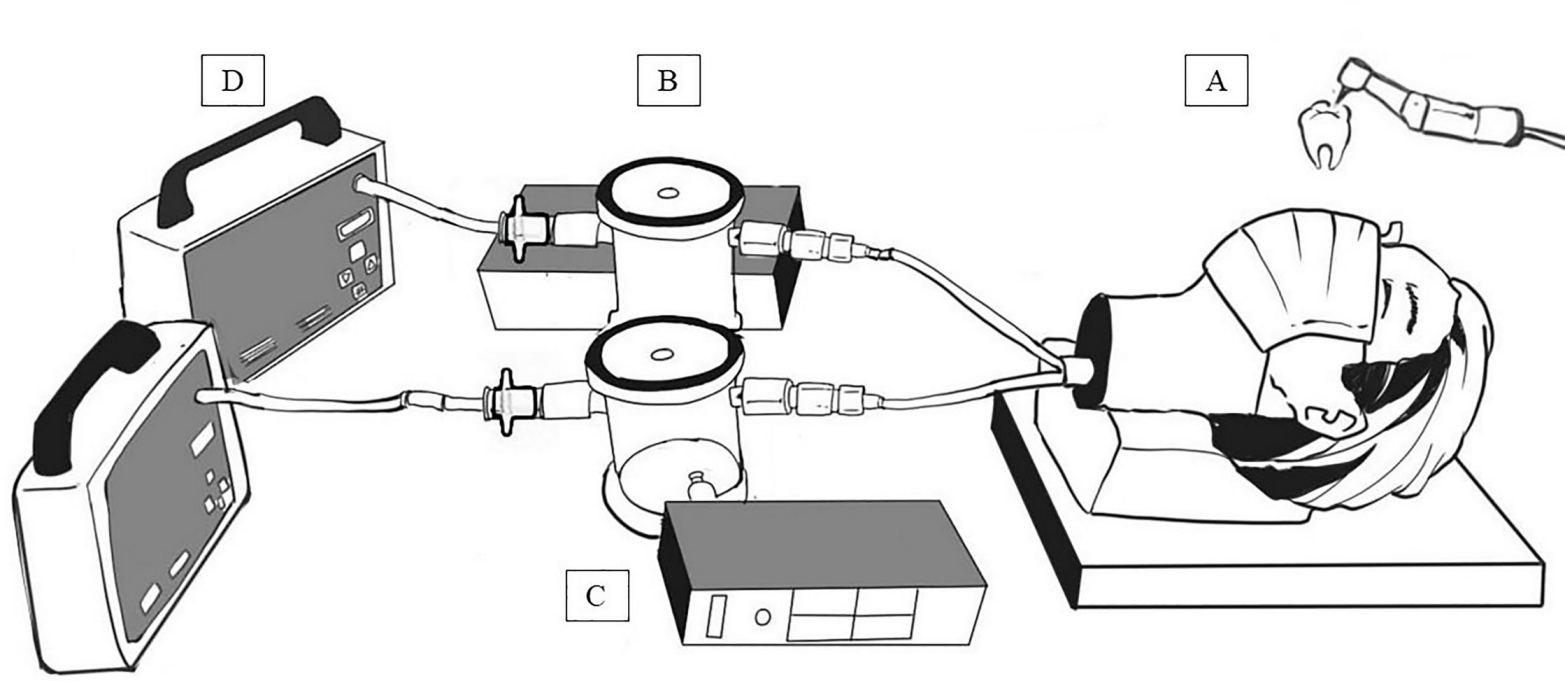
Experimental model system for suspended particles measurement when drilling and grinding teeth. A: head-form model, B: collection chamber, C: particle counter, D: pumping system.

A head-form model was used to simulate a dental medical employee wearing a protective mask (Fig 1). Mobile dental treatment equipment (DCII manual control, Joyo Welldent Company, Taiwan) was used to drill and grind all extracted teeth. The rotary speed and work efficiency of the high-speed instrument was similar to that used clinically (e.g., 350,000–400,000 cycles per minute) and 50–60 LPM of water was produced to reduce the temperature of the drilling surface.

### Testing the efficiency of protective medical masks and the central vacuum system

The pumping system flow rate was set to 8.5 LPM to simulate nasal breathing in the human body when wearing close-fitting protective masks. A close fit achieved with tape fittings along the edges of the mask and the bridge of the nose to ensure the mask fitted to the face completely. Two kinds of protective masks were used to compare the efficiency of PM removal via the central vacuum system and medical surgical masks (SafeMask® Surgical Tie-On Mask, Medicom, U.S.A.), as well as N95 masks (N95 Particulate Respirator-SH2950, San Huei, Taiwan).

This study detected the indoor background particle concentrations before starting the experiment of drilling and grinding the teeth. All teeth-drilling processes took two minutes and then collected air samples under the central vacuum system was open or closed combined with wearing personal medical masks conditions. The suction flow (28.5 LPM) of the central vacuum system was according to clinical practice. In this study, 20 teeth were drilled and a new grinding bur was used for each tooth. Particle size and concentration were assessed at the distance of 15 cm from the treated teeth, as was the efficiency of the central vacuum equipment and the protective masks for reducing levels of PMs.

A portable particle counter (model 1.109, Grimm Labortechnik Ltd., Ainring, Germany) was used to evaluate the size of particles produced by tooth drilling and grinding. This consisted of a 31–degree size channel and monitored for particles between 0.265 μm and 34 μm in size (defined as total suspended particles, TSP). The recordings of the particle counter were taken every six seconds. The concentrations of PM_≥0.5_ (aerodynamic diameter, da ≥ 0.5 μm), PM_10_ (da ≤ 10 μm), PM_2.5_ (da ≤ 2.5 μm), PM_2.5–10_ (2.5 < da ≤ 10 μm), and PM_1_ (da ≤ 1 μm) were recorded. A hand-held Nano Analyzer (Nanotracer, Philips Aerasense, Eindhoven, NL), which measures 10–300 nm (0.01–0.3 μm) particles (PM_0.01–0.3_), was also used to monitor changes in the concentration of nanoparticles. This study defined the nanoparticles sized 10–300 nm as ultrafine particles. Moreover, the particle concentrations were measured inside (I) and outside (O) the masks with or without central vacuum system while drilling the teeth, and the I/O ratios were calculated in this study.

### Statistical analyses

Each experiment was repeated five times. This study used SPSS 22.0 software (SPSS, Chicago, Illinois) for statistical analyses and the significance level was set to 0.05. GraphPad Prism 6.0 software (GraphPad Software, Inc., USA) was used to graph data. The Wilcoxon signed-rank test was used to determine the removal efficiency of particles of various sizes (PM_≥0.5_, PM_10_, PM_2.5_, PM_1_, and PM_0.01–0.3_) with or without central vacuum during molar grinding. The Mann-Whitney U test was used to compare differences in the PM levels of drilling teeth and indoor air background, and in filtration efficiency of medical surgical masks and N95 masks.

## Results

**Fig 2:**
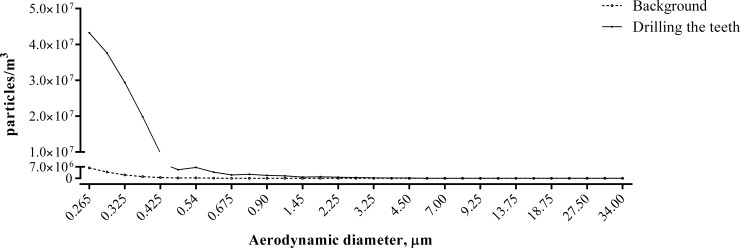
Particle size distributions of drilling the teeth and non-drilling the teeth in non-suction conditions.

**Fig 3:**
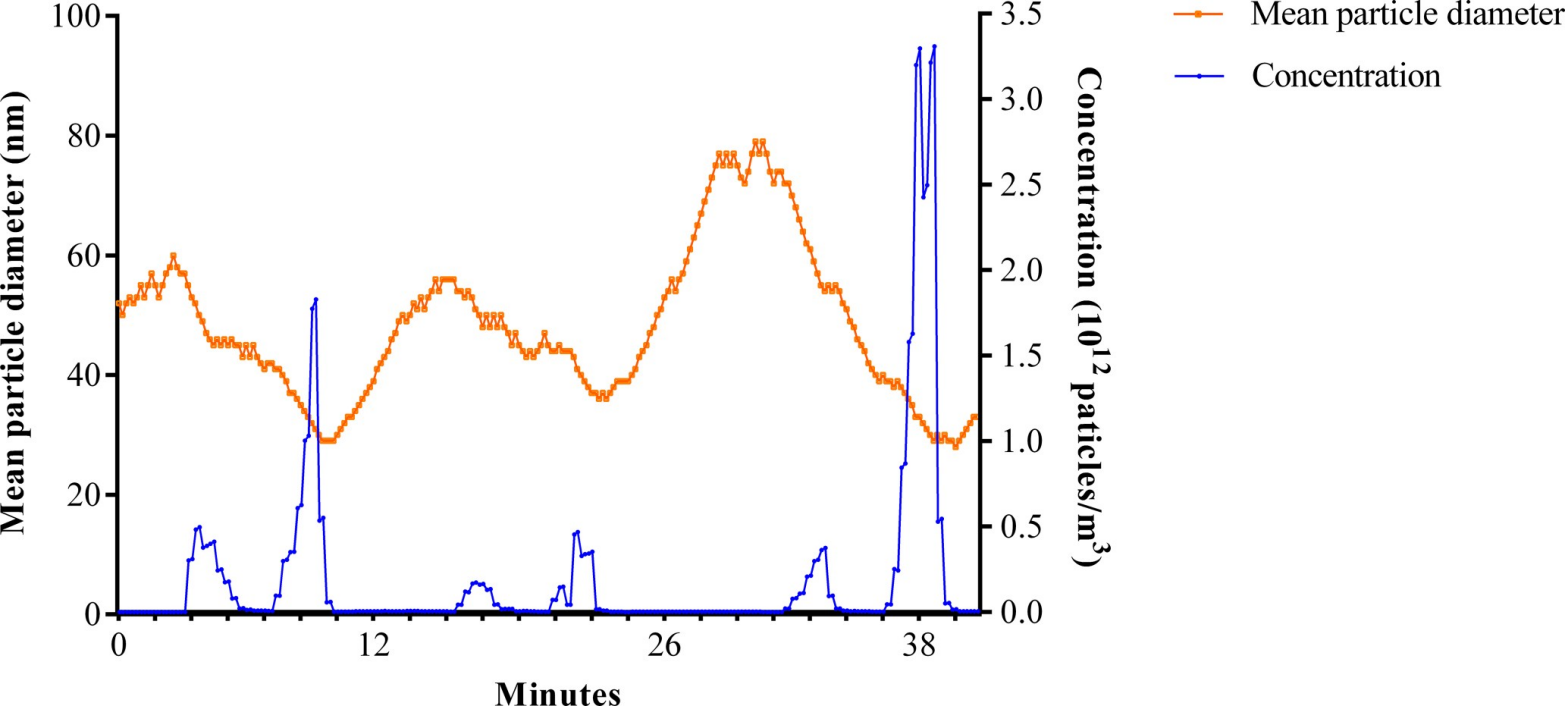
Particle size and concentration distributions of ultrafine particles generated from drilling teeth procedures.

The present study demonstrated that the number concentration of total PM produced by grinding teeth (1.72×10^8^ particles/m^3^) was significantly higher than the indoor background concentration (1.49×10^7^ particles/m^3^) (*P* = 0.001) (Fig 2). During tooth drilling and grinding without vacuum, 0.85% of TSP were PM_2.5–10_, 99.15% of TSP were PM_2.5_, and 96.78% of TSP were PM_1_. Surprisingly, 97.62% of PM_2.5_ was PM_1_. It meant that the particles produced by drilling and grinding teeth were almost entirely PM_1_. Furthermore, the aerodynamic diameter of most particles measured by a Nano analyzer was below 70 nm and the average particle diameter was 53.68 nm. In addition, the average number concentration of ultrafine particles was 2.1x10^11^ particles/m^3^, while drilling teeth in the absence of a vacuum system (Fig 3).

The present study also assessed the effectiveness of a central vacuum system for PM removal. The median number concentration of PM_≥0.5_ (1.83x10^7^ particles/m^3^) without use of a vacuum system was significantly higher than that detected with use of a vacuum system (9.54x10^6^ particles/m^3^, *P* < 0.01) (Table 1). Similar results were found with the number concentrations of PM_10_, PM_2.5_, and PM_1_ that produced by tooth drilling. The mean vacuum suction efficiency was 35.74% for PM_≥0.5_, 35.41% for PM_10_, 35.47% for PM_2.5_, and 35.58% for PM_1_ in this study. However, no difference was found in the number concentration of PM_0.01–0.3_ generated by drilling and grinding teeth with or without use of a vacuum system.

Additionally, the mass concentrations of PM_≥0.5_ (without vacuum system: 112.05 μg/m^3^, with vacuum system: 44.17 μg/m^3^) and PM_10_ (without vacuum system: 80.32 μg/m^3^, with vacuum system: 25.40 μg/m^3^) did not differ with or without vacuum system use during tooth drilling. The median mass concentration of PM_2.5_ detected without vacuum system use was 20.48 μg/m^3^ higher than that detected with vacuum status (9.57 μg/m^3^, *P* < 0.01). The median mass concentration of PM_1_ detected without vacuum system use (7.87 μg/m^3^) was also significantly higher than that detected with vacuum system use (4.18 μg/m^3^, *P* < 0.01).

**Table 1:** Comparison of particle concentrations under drilling the teeth without/with suction conditions.

	Without suction (n = 10)		With suction (n = 10)		*P* value
Number concentration, particles/m^3^					
PM_≥0.5_	1.83x10^7^	(1.40x10^7^–2.18x10^7^)	9.54x10^6^	(6.29x10^6^–1.57x10^7^)	<0.001
PM_10_	1.41x10^5^	(1.24x10^5^–1.70x10^5^)	8.35x10^4^	(6.32x10^4^–1.30x10^5^)	<0.001
PM_2.5_	1.39x10^5^	(1.23x10^5^–1.69x10^5^)	8.32x10^4^	(6.29x10^4^–1.28x10^5^)	<0.001
PM_1_	1.35x10^5^	(1.20x10^5^–1.65x10^5^)	8.18x10^4^	(6.29x10^7^–1.23x10^5^)	<0.001
Mass concentration, μg/m^3^					
PM_≥0.5_	112.05	(77.98–132.85)	44.17	(26.86–126.42)	0.050
PM_10_	80.32	(67.55–114.58)	25.40	(21.47–126.56)	0.109
PM_2.5_	20.48	(16.59–25.79)	9.57	(6.72–26.21)	0.001
PM_1_	7.87	(5.94–8.08)	4.18	(2.94–7.72)	<0.001

Data were presented as median (25–75 percentiles).

When teeth were ground under the condition without vacuum but in the presence of surgical and N95 masks, the analytical results indicated that we found no difference in the concentration of all sizes of PMs. The ratio of PM concentrations inside and outside the mask did not change across categories, including PM_≥0.5_ (Surgical mask: 0.80, N95 mask: 1.34), PM_10_ (Surgical mask: 0.82, N95 mask: 1.37), PM_2.5_ (Surgical mask: 0.82, N95 mask: 1.37), and PM_1_ (Surgical mask: 0.82, N95 mask: 1.37) (Table 2). Additionally, when both a vacuum system and surgical mask (PM_≥0.5_: 1.18, PM_10_: 1.11, PM_2.5_: 1.11, and PM_1_: 1.10) or N95 mask (PM_≥0.5_: 1.36, PM_10_: 1.26, PM_2.5_: 1.26, PM_1_: 1.26) were used, the ratio of PM concentrations inside and outside the mask had also no difference between the two kinds of masks.

**Table 2:** The I/O ratios of the concentrations of suspended particulate matter in the surgical masks and N95 masks under without/with suction conditions.

	Surgical mask	N95 Mask	*P* value
Without suction			
PM_≥0.5_,	0.80	1.34	0.173
PM_10_	0.82	1.37	0.116
PM_2.5_	0.82	1.37	0.117
PM_1_	0.82	1.37	0.117
With suction			
PM_≥0.5_	1.18	1.36	0.602
PM_10_	1.11	1.26	0.465
PM_2.5_	1.11	1.26	0.465
PM_1_	1.10	1.26	0.465

## Discussion

The drilling and grinding of teeth thus increased suspended particle concentrations in the dental environment. Suspended particles may derive from sources beyond teeth, however, leading to even higher concentrations of PM in dental offices while tooth grinding is ongoing. Nanoparticles are also released during abrasive dental procedures such as reshaping and grinding of ceramics, metals, and polymer materials [7]. A recent U.S. study found that indoor particles in a dentistry clinic were mainly composed of 6500 nm in size particles, with ultrafine particles (< 100 nm in size) accounting for 67% of all particles present [19]. Furthermore, a previous study reported that the breathing zone of both the dentist and patient included a high concentration of nanoparticles when composite resin grinding as ongoing [8]. Here, we demonstrate that the particles produced by tooth drilling were almost entirely PM_1_ particles. These fine particles may enter the respiratory system alveoli, penetrating deep into the lungs [20].

The mechanisms underlying aerosol transfusion include inertial impaction, gravitational sedimentation, and Brownian diffusion. Inertial impaction primarily occurs among particles with a diameter larger than 6 μm. When air flows through the upper respiratory tract or airway bifurcation, abrupt directional changes may prevent larger particles from continuing to travel in the direction of airflow, thus depositing on the airway walls. The gravity sedimentation primarily occurs among 26 μm particles. When air flows into the small airway, a decreased airflow velocity, gravity, and sedimentation causes particles to stick to the airway walls. Brownian motion primarily affects particles less than 2 μm in diameter. When airflow velocity is low, irregular and random collisions occur between particles, turning them into alveolar sedimentation [20]. This suggests that high concentrations of PMs, produced by drilling and grinding of the teeth, as revealed by the present study, may pose a respiratory health risk to dental personnel.

The present study found that the central vacuum system led to approximately 36% reductions in aerosolized particles generated by drilling and grinding teeth. However, this system had no influence on the size or concentration of nanoparticles produced by tooth drilling and grinding. Another study reported that high-volume evacuation may reduce the concentration of aerosols during dental procedures [12], a result which is inconsistent with those presented here. Further studies should evaluate the relationship between vacuum flow and changes of PM / bioaerosol concentrations to prevent hazardous exposure of medical personnel and patients.

We found no significant filtration efficiency differences between the two kinds of masks tested here in terms of detected PM size during grinding with or without a vacuum system. Furthermore, we found an average filtration efficiency of the medical surgical masks of only approximately 20%. However, the aerodynamic diameter of PM generated by tooth grinding was almost below 70 nm, per the ultrafine particle analyzer used here. Therefore, the N95 and medical surgical masks used here do not offer a sufficient protection efficiency to prevent nanoparticle passage of the airways. A previous study that assessed the dry residue weight and filter efficiency of two types of surgical face masks (1818 Tie-On surgical mask and 1942 FB fluid resistant molded surgical mask) and a personal respirator (1862 health care particulate respirator), and found that a certified personal respirator may be more effective in filtering efficiency than high-quality surgical masks [21].

Particles in a dental office may be generated by a number of instruments, including air-turbine handpieces, low-speed handpieces, ultrasonic scalers, bicarbonate polishers, and polishing cups, as well as drilling and air sprays inside the oral cavity [6]. Strong evidence suggests that these procedures result in particle concentrations above background levels [7]. A previous study revealed that the highest submicrometer particle concentrations detected during dental grinding were 16 times higher than indoor background concentrations [13]. Dental drilling procedures also produce higher concentrations of PM_<0.5_ than PM_>0.5_ [6]. Inhalation of these smaller particles may pose significant human health risks, especially when particles are small enough to penetrate deep into the lungs [6]. It deserves careful consideration to remove suspended particles during dental procedures by using effective vacuum system and select optimal high-level personal face masks for dentists and other medical staffs.

A limitation of the present study was that effectively quantifying the actual exposure of dental staff was difficult. However, we found that high PM_1_ concentrations were detected when drilling and grinding teeth. The vacuum efficiency of the central vacuum system and the filtration efficiency offered by protective masks have limited efficacy. Therefore, some strategies would suggested preventing fine and ultrafine particles exposure, such as promoting awareness of occupational health and safety, increasing ventilation rates in the work area, using local exhaust ventilation, and wearing high-level personal protective equipment.

In conclusion, the particles produced by tooth drilling and grinding were almost entirely PM_1_. A significant difference was found in the concentration of fine particles with the use of a vacuum system but not in the concentration of ultrafine particles. No significant differences in I/O ratios for either of the two kinds of protective mask tested here were found in terms of measured particle diameters, with or without the use of a vacuum system.

## Supporting information

S1 Data (XLSX)
